# A comparison of two assessment tools used in overviews of systematic reviews: ROBIS versus AMSTAR-2

**DOI:** 10.1186/s13643-021-01819-x

**Published:** 2021-10-25

**Authors:** R. Perry, A. Whitmarsh, V. Leach, P. Davies

**Affiliations:** 1 National Institute for Health Research Bristol Biomedical Research Centre, University Hospitals Bristol and Weston NHS Foundation Trust and University of Bristol, Bristol, UK; 2 The National Institute for Health Research Applied Research Collaboration West (NIHR ARC West) at University Hospitals Bristol NHS Foundation Trust, Bristol, UK; 3 Population Health Sciences, Bristol Medical School, University of Bristol, Bristol, UK

**Keywords:** AMSTAR-2, ROBIS, Systematic reviews, Methodological quality, Risk of bias

## Abstract

**Background:**

AMSTAR-2 is a 16-item assessment tool to check the quality of a systematic review and establish whether the most important elements are reported. ROBIS is another assessment tool which was designed to evaluate the level of bias present within a systematic review. Our objective was to compare, contrast and establish both inter-rater reliability and usability of both tools as part of two overviews of systematic reviews. Strictly speaking, one tool assesses methodological quality (AMSTAR-2) and the other assesses risk of bias (ROBIS), but there is considerable overlap between the tools in terms of the signalling questions.

**Methods:**

Three reviewers independently assessed 31 systematic reviews using both tools. The inter-rater reliability of all sub-sections using each instrument (AMSTAR-2 and ROBIS) was calculated using Gwet’s agreement coefficient (AC_1_ for unweighted analysis and AC_2_ for weighted analysis).

**Results:**

Thirty-one systematic reviews were included. For AMSTAR-2, the median agreement for all questions was 0.61. Eight of the 16 AMSTAR-2 questions had substantial agreement or higher (> 0.61). For ROBIS, the median agreement for all questions was also 0.61. Eleven of the 24 ROBIS questions had substantial agreement or higher.

**Conclusion:**

ROBIS is an effective tool for assessing risk of bias in systematic reviews and AMSTAR-2 is an effective tool at assessing quality. The median agreement between raters for both tools was identical (0.61). Reviews that included a meta-analysis were easier to rate with ROBIS; however, further developmental work could improve its use in reviews without a formal synthesis. AMSTAR-2 was more straightforward to use; however, more response options would be beneficial.

## Background

Systematic reviews have become a fundamental part of evidence-based medicine; they are considered the highest form of evidence as they synthesise all available evidence on a given topic [1]. Many will also combine data to give an overall effect estimate using a meta-analysis. However, the quality and standard of reviews varies considerably. If this is not understood, or in some way established, the results of many reviews might be overstated. Quality assessment tools have been developed to assess such variation in standards.

One previously heavily cited tool is the Assessment of Multiple Systematic Reviews (AMSTAR) scale [2] which has been widely used since its development in 2007. This scale was shown to be both reliable and valid [3]. However, it came under criticism for some issues with its design. It was argued by Burda et al. [4] that AMSTAR was lacking in some key constructs, in particular, the confidence in the estimates of effect. It also lacks an item to assess subgroup and sensitivity analysis. Further criticisms include issues such as the inclusion of foreign language papers as “grey literature” and the idea that the items can often partially but not fully meet the criteria was highlighted. Also, each item was not weighted evenly and there is a lack of overall score, which became problematic when trying to compare scores. Thus, an upgraded version (AMSTAR-2) was developed in 2017*.* The new version promised to simplify the response categories, align the definition of research questions with the PICO (population, intervention, control group, outcome) framework, seek justification for the review authors’ selection of different study designs (randomised and non-randomised) and included numerical rating scales for inclusion in systematic reviews, seek reasons for exclusion of studies from the review, and determine whether the review authors had made a sufficiently detailed assessment of risk of bias for the included studies and whether risk of bias was considered adequately during statistical pooling and when interpreting the results [5].

A second novel assessment tool that has undergone rigorous development was published in 2016 (Risk of Bias in Systematic reviews [ROBIS [6]]). It aimed to provide a thorough and robust assessment of the level of bias within the systematic review.

### Description of the assessment tools

#### Assessment of multiple systematic reviews (AMSTAR-2)

The main aim of the AMSTAR-2 is a tool to assess the methodological quality of the review. It is made up of 16 items in total and has simpler response categories than the original AMSTAR version. Some sections are considered by the authors to be *critical domains,* which can be used for determining an overall score (see Appendix, Table 12 for more information on the critical domains). AMSTAR-2 is intended for assessing effectiveness. The tool can also be applied to reviews of both randomised and non-randomised studies.

#### ROBIS tool

The main aim of the ROBIS tool is to evaluate the level of bias present within a systematic review. The tool is made up of three distinct phases. Firstly, there is an optional first phase to assess the applicability of the review to the research question of interest. The second phase is made up of 20-items within four main domains: study eligibility criteria, identification and selection of studies, data collection and study appraisal, synthesis and findings. This phase is to identify concerns about the review conduct. Each domain has signalling questions and ends with a judgement of concerns of each domain (low, high or unclear). There is also a third phase consisting of three signalling questions to enable an overall assessment of bias rating to be given. ROBIS has a wide application and is intended for assessing effectiveness, diagnostic test accuracy, prognosis and aetiology [6].

### Previous research

Due to the novelty of both tools, there is limited available literature comparing them; however, some work has been recently published.

One review team [7, 8] compared all three tools (AMSTAR, AMSTAR-2 and ROBIS), applying them to reviews that reported both randomised and non-randomised trials. The inter-rater reliability between four raters’ across 30 systematic reviews was analysed. Minor differences were found between AMSTAR-2 and ROBIS in the assessment of systematic reviews including a mix of study type. On average, the inter-rater reliability (IRR) was higher for AMSTAR-2 compared to ROBIS. They assumed that scoring ROBIS would take more time in general, and it was always applied after AMSTAR-2, but in fact the mean time for scoring AMSTAR-2 was slightly higher than for ROBIS (18 vs. 16 min), with huge variation between the reviewers. They also reported that some signalling questions in ROBIS were judged to be very difficult to assess.

### Aim

The overarching aim of our work is to add to the literature and make a further comparison of both assessment tools in two overviews of reviews. Our team had previously completed two overviews on complementary and alternative medicine (CAM) therapies for two hard-to-treat conditions. One overview evaluated systematic reviews of various CAM therapies for fibromyalgia (FM) [9], and the other evaluated systematic reviews of CAM therapies for infantile colic [10].

### Objectives

Due to some of the challenges we had using both tools in our overview of reviews work, we planned a formal assessment of both tools by completing the following comparisons and evaluations:

1. To compare the content of the tools
2. To compare the percentage agreement (IRR)
3. To assess the useability/user experience of both tools.

## Methods

Two overviews of reviews were conducted by our team [9, 10]. The first reviewed CAM for fibromyalgia and assessed the included reviews using both the original AMSTAR tool [2] and ROBIS [6]. This review was published in 2016, prior to the development and publication of AMSTAR-2 [5]. Here, we reported on 15 systematic reviews of CAM for fibromyalgia, published between 2003 and 2014 which assessed several CAM therapies. Eight of the reviews included a quantitative synthesis.

We subsequently completed a second overview of reviews of CAM for infantile colic published in 2019 [10]. Here, we used the new AMSTAR-2 tool alongside ROBIS. We reported on 16 systematic reviews of CAM for colic, published between 2011 and 2018. The reviews investigated several CAM therapies, 12 of which included a quantitative synthesis.

We later returned to the fibromyalgia review papers and reassessed them all using the AMSTAR-2 scale, for consistency. This results in a total comparison of 31 reviews. The reviewers were not strict about the order of ratings.

### Assessment of methodological quality/bias of the included reviews

Three reviewers (RP, VL, PD) independently assessed each systematic review using both tools. Any reported meta-analyses were checked by a statistician experienced in meta-analyses (CP). The final score was agreed after discussion between the authors.

### Data-analysis

Gwet’s AC statistic was used to calculate inter-rater reliability (IRR) [11]. Gwet’s AC2 is a weighted statistic which allows for “partial agreement” between ordinal categories. Therefore, Gwet’s AC2 was used to calculate IRR (using linear weights) for AMSTAR-2 questions with options “no”, “partial yes” and “yes” (questions 2, 4, 7, 8, 9). Gwet’s AC1 is an unweighted statistic which measures full agreement only. Gwet’s AC1 was used for all other AMSTAR-2 questions.

All signalling questions for ROBIS were analysed using Gwet’s AC2 with linear weights where “no”, “probably no”, “probably yes” and “yes” were recoded as 1–4. As mentioned above, Gwet’s AC2 is a weighted statistic which allows for “partial agreement” between ordinal categories. Ratings of “no information” were treated as missing. Gwet’s AC1 was used for ROBIS domains. Agreement for AMSTAR-2 and ROBIS was classified as “poor” (≤ 0.00), “slight” (0.01–0.20), “fair” (0.21–0.40), “moderate” (0.41–0.60), “substantial” (0.61–0.80), and “almost perfect” (0.81–1.00), following accepted criteria [12]. All analyses were completed using Stata 16 (StataCorp. 2019; Stata Statistical Software).

## Results

Our first objective was to compare the content of the tools (see Table 1). Any overlaps and discrepancies between the two scales are identified. Overall, we found considerable overlap on the signalling questions. However, ROBIS does not assess whether there is a comprehensive list of studies (both included and excluded) or whether any conflicts of interest were declared (both at the individual trial level and for the reviews), as these are considered issues of methodology quality rather than bias. AMSTAR-2 also assessed possible conflicts of interest, which is not assessed in ROBIS, despite being a potential risk of bias. However, the section on synthesis was given more in-depth consideration in ROBIS tool.

**Table 1:** A comparison of the content of the two tools (AMSTAR-2 and ROBIS)

Criteria	AMSTAR-2	ROBIS
**Eligibility criteria**	1. Did the research questions and inclusion criteria for the review include the components of PICO? 2. Did the report of the review contain an explicit statement that the review methods were established prior to the conduct of the review and did the report justify any significant deviations from the protocol? 3. Did the review authors explain their selection of the study designs for inclusion in the review?	1.1 Did the review adhere to pre-defined objectives and eligibility criteria? 1.2 Were the eligibility criteria appropriate for the review question? 1.3 Were eligibility criteria unambiguous? 1.4 Were all restrictions in eligibility criteria based on study characteristics appropriate (e.g., date, sample size, study quality, outcomes measured)? 1.5 Were any restrictions in eligibility criteria based on sources of information appropriate (e.g., publication status or format, language, availability of data)?
**Study selection and Data extraction**	5. Did the review authors perform study selection in duplicate? 6. Did the review authors perform data extraction in duplicate?	2.5 Were efforts made to minimise error in selection of studies? 3.1 Were efforts made to minimise error in data collection? 3.3 Were all relevant study results collected for use in the synthesis?
**Literature search**	4. Did the review authors use a comprehensive literature search strategy?	2.1 Did the search include an appropriate range of databases/electronic sources for published and unpublished reports? 2.3 Were the terms and structure of the search strategy likely to retrieve as many eligible studies as possible? 2.4 Were restrictions based on date, publication format, or language appropriate?
**Grey literature**	NA	2.2 Were methods additional to database searching used to identify relevant reports?
**List of studies**	7. Did the review authors provide a list of excluded studies and justify the exclusions?	N/A
**Characteristics of studies**	8. Did the review authors describe the included studies in adequate detail?	3.2 Were sufficient study characteristics available for both review authors and readers to be able to interpret the results?
**Quality assessment**	9. Did the review authors use a satisfactory technique for assessing the risk of bias (RoB) in individual studies that were included in the review?	3.4 Was risk of bias (or methodological quality) formally assessed using appropriate criteria? 3.5 Were efforts made to minimise error in risk of bias assessment?
**Synthesis of the findings**	N/A N/A 11. If meta-analysis was performed did the review authors use appropriate methods for statistical combination of results? 12. If meta-analysis was performed, did the review authors assess the potential impact of RoB in individual studies on the results of the meta-analysis or other evidence synthesis?	4.1 Did the synthesis include all studies that it should? 4.2 Were all pre-defined analyses reported or departures explained? 4.3 Was the synthesis appropriate given the nature and similarity in the research questions, study designs and outcomes across included studies? 4.6 Were biases in primary studies minimal or addressed in the synthesis?
**Heterogeneity**	14. Did the review authors provide a satisfactory explanation for, and discussion of, any heterogeneity observed in the results of the review? 15. If they performed quantitative synthesis did the review authors carry out an adequate investigation of publication bias (small study bias) and discuss its likely impact on the results of the review?	4.4 Was between-study variation (heterogeneity) minimal or addressed in the synthesis? 4.5 Were the findings robust, e.g., as demonstrated through funnel plot or sensitivity analyses?
**Interpretation**	13. Did the review authors account for RoB in individual studies when interpreting/ discussing the results of the review?	A. Did the interpretation of findings address all of the concerns identified in Domains 1 to 4? B. Was the relevance of identified studies to the review’s research question appropriately considered? C. Did the reviewers avoid emphasising results on the basis of their statistical significance?
**Conflict of interest**	10. Did the review authors report on the sources of funding for the studies included in the review? 16. Did the review authors report any potential sources of conflict of interest, including any funding they received for conducting the review?	N/A N/A

Signalling questions are in a different order to line up the criteria from both tools. *N/A* not assessed

### Section 2: Comparison of the inter-rater reliability of the tools

#### AMSTAR-2

The consensus results for AMSTAR-2 of both fibromyalgia and colic overviews can be found in Table 2. We report on 15 systematic reviews of CAM for fibromyalgia and found all but one review [13] rated as having critically low confidence in the results (see Appendix, Table 15 for scoring information). This was the only Cochrane review included in the FM overview. We also report on 16 systematic reviews of CAM for colic. Most were rated as having critically low confidence in the results, 4 were rated as low and 1 (a Cochrane review) was considered to have high confidence in the results. The comparison of the ratings for each review can be found in the Appendix (see Tables 9, 10, 13, and 14). There were a greater number of discrepancies between the overall risk of bias and quality ratings in the fibromyalgia reviews. The overall risk of bias/quality ratings was more consistent in the colic reviews.

**Table 2:** Agreed results of AMSTAR-2 for fibromyalgia

Author (date), CAM	1. Were PICO components listed?	*2. Protocol reported? Any deviations justified?*	3. Study design justified?	*4. Comprehensive literature search?*	5. Was study selection performed in duplicate?	6. Was data extraction performed in duplicate?	*7. List of excluded studies? Were these justified?*	8. Characteristics of studies provided in detail?
**Fibromyalgia**								
**Multiple cam therapies**								
Holdcraft 2003 [14]	No	*No*	No	*No*	No	No	*No*	No
Baronowsky 2009 [15]	No	*No*	Yes	*No*	No	No	*No*	No
Terhorst 2011, 2012 [16, 17]	Yes	*No*	No	*No*	Yes	No	*No*	No
De Silva 2010 [18]	No	*No*	No	*No*	Yes	Yes	*No*	No
**Homoeopathy**								
Perry 2010 [19]	Yes	*No*	No	*PY*	Yes	Yes	*No*	PY
Boehm 2014 [20]	No	*No*	No	*PY*	Yes	Yes	*No*	Yes
**Chiropractic treatment**								
Ernst 2009 [21]	Yes	*No*	No	*No*	No	Yes	*No*	No
**Acupuncture**								
Mayhew and Ernst 2007 [22]	No	*No*	No	*PY*	No	Yes	*No*	PY
Daya 2007 [23]	No	*No*	No	*PY*	No	No	*No*	PY
Langhorst 2010 [24]	Yes	*No*	No	*PY*	Yes	Yes	*No*	Yes
Martin-Sanchez 2009 [25]	Yes	*No*	No	*PY*	No	No	*No*	No
Cao 2013 [26]	Yes	*No*	No	*PY*	yes	Yes	*No*	Yes
Deare 2013 [13]	Yes	*Yes*	No	*Yes*	Yes	Yes	*Yes*	Yes
Yang 2014 [27]	Yes	*No*	No	*PY*	Yes	Yes	*No*	No
**Herbal medicines**								
de Souza Nascimento 2013 [28]	Yes	*No*	yes	*No*	No	Yes	*No*	PY
**Colic**								
**Multiple cam therapies**								
Perry 2011 [29]	Yes	*PY*	No	*PY*	PY	Yes	*No*	Yes
Bruyas-Bertholon 2012 [30]	No	*No*	No	*No*	No	No	*No*	PY
Harb 2016 [31]	Yes	*No*	No	*No*	Yes	Yes	*No*	PY
Gutierrez-Castrellon 2017 [32]	Yes	*No*	No	*No*	No	No	*No*	No
**Manipulation therapies**								
Dobson 2012 [33]	Yes	*Yes*	No	*Yes*	Yes	Yes	*Yes*	Yes
Gleberzon 2012 [34]	No	*No*	No	*PY*	No	Yes	*No*	PY
Carnes 2017 [35]	No	*PY*	No	*PY*	Yes	Yes	*No*	PY
**Acupuncture**								
Skejeie 2018 [36]	Yes	*PY*	No	*Yes*	Yes	Yes	*No*	Yes
**Herbal medicines**								
Anheyer 2017 [37]	No	*No*	No	*No*	No	Yes	*No*	Yes
**Probiotics**								
Sung 2013 [38]	Yes	*No*	No	*No*	Yes	Yes	*Yes*	PY
Anabrees 2013 [39]	Yes	*PY*	No	*PY*	No	Yes	*No*	Yes
Urbanska 2014 [40]	Yes	*No*	No	*PY*	No	No	*No*	PY
Xu 2015 [41]	No	*No*	No	*PY*	Yes	Yes	*No*	Yes
Schreck Bird 2017 [42]	Yes	*No*	No	*No*	Yes	No	*No*	Yes
Dryl 2018 [43]	Yes	*No*	No	*No*	No	No	*No*	PY
Sung 2018 [44]	Yes	*PY*	No	*PY*^*b*^	No	No	*No*	No

*9. Risk of bias assessed?*	10. Sources of funding of included studies?	*11. Methods used to combine the findings of studies appropriate? Test on heterogeneity?*	12. If meta-analysis performed was RoB accounted for?	*13. Was RoB discussed in individual studies?*	14. Was there discussion of any heterogeneity observed in the results?	*15. If a quantitative synthesis, was publication bias investigated and discussed in relation to the results?*	16. Reviewers’ conflict of interests stated?	***Confidence in the review***
**Fibromyalgia**								
**Multiple cam therapies**								
*PY*	No	*No MA*	No MA	*Yes*		*No MA*	No	***CL***
*No*	No	*No MA*	No MA	*Yes*	No	*No MA*	No	***CL***
*Yes*	No	*No*	No	*No*	No	*No*	Yes	***CL***
*No*	No	*No MA*	No MA	*Yes*	Yes	*No MA*	Yes	***CL***
**Homoeopathy**								
*Yes*	No	*No MA*	No MA	*Yes*	Yes	*No MA*	Yes	***CL***
*Yes*	No	*Yes*	No	*No*	Yes	*No*	Yes	***CL***
**Chiropractic treatment**								
*No*	No	*No MA*	No MA	*Yes*	No	*No MA*	Yes	***CL***
**Acupuncture**								
*No*	NO	*No MA*	No MA	*Yes*	Yes	*No MA*	Yes	***CL***
*PY*	No	*No MA*	No MA	*No*	No	*No MA*	No	***CL***
*Yes*	No	*Yes*	Yes	*Yes*	Yes	*No*	No	***CL***
*No*	No	*No*	No	*No*	Yes	*No*	Yes	***CL***
*Yes*	No	*Yes*	No	*Yes*	Yes	*Yes*	Yes	***CL***
*Yes*	Yes	*Yes*	Yes	*Yes*	Yes	*No*	Yes	***Low***
*Yes*	No	*No*	No	*No*	No^a^	*Yes*	No	***CL***
**Herbal medicines**								
*Yes*	No	*No MA*	No MA	*No*	No	*No MA*	Yes	***CL***
**Colic**								
**Multiple cam therapies**								
*Yes*	No	*No MA*	No MA	*Yes*	No	*No MA*	Yes	***Low***
*PY*	No	*No MA*	No MA	*No*	No	*No MA*	No	***CL***
*Yes*	No	*Yes*	Yes	*Yes*	No	*Yes*	Yes	***CL***
*No*	No	*No*	No	*Yes*	No	*Yes*	No	***CL***
**Manipulation therapies**								
*Yes*	No	*Yes*	Yes	*Yes*	No^a^	*Yes*	Yes	***High***
*No*	No	*No MA*	No MA	*Yes*	No	*No MA*	No	***CL***
*Yes*	No	*No*	No	*No*	No	*No*	No	***CL***
**Acupuncture**								
*Yes*	No	*Yes*	Yes	*Yes*	Yes	*No*	No^d^	***Low***
**Herbal medicines**								
*Yes*	No	*No MA*	No MA	*No*	No	*No MA*	Yes	***CL***
**Probiotics**								
*Yes*	No	*Yes*	No	*Yes*	No	*No*	Yes	***CL***
*Yes*	No	*Yes*	No	*Yes*	Yes	*No*	Yes	***Low***
*Yes*	Yes	*No*	No	*No*	No	*No*	No	***CL***
*Yes*	No	*Yes*	No	*No*	No	*No*	Yes	***CL***
*Yes*	No	*No*	No	*No*	No	*No*	No	***CL***
*Yes*	No	*No*	No	*No*	No	*No*	No	***CL***
*Yes*	No	*Yes*	Yes	*Yes*	Yes	*Yes*	No^c^	***Low***

*CL* critically low, *PY* partial yes, *MA* meta-analysis, *PICO* participants, intervention, comparator, outcomes, *RoB* risk of bias

^a^Too few studies to perform a test of heterogeneity

^b^Not fully searched and search conducted Dec 2014

^c^Conflict of interest occurred but no indication of how it was dealt with

^d^All included studies were by the author team but did not indicate how this was dealt with

Italicised columns represent the critical domains (see Appendix, Table 15)

##### Results of inter-rater reliability analysis for AMSTAR-2

A summary of the inter-rater reliability [IRR] for AMSTAR-2 can be found in Table 3. Seven questions that relate to critical domains were identified by Shea et al. [5]; more information about these domains can be found in Appendix (Table 15).

**Table 3:** The inter-rater agreement between the three raters for AMSTAR-2

Question	Number of studies	Gwet’s AC1/Gwet’s AC2	95% CI
1	31	0.69	0.48, 0.91
*2*	*31*	*0.93*	*0.85, 1.00*
3	31	0.55	0.30, 0.80
*4*	*31*	*0.66*	*0.51, 0.81*
5	31	0.70	0.47, 0.94
6	31	0.60	0.35, 0.86
*7*	*31*	*0.97*	*0.94, 1.00*
8	31	0.39	0.21, 0.56
*9*	*31*	*0.65*	*0.46, 0.84*
10	31	0.84	0.67, 1.00
*11*	*19*	*0.54*	*0.19, 0.89*
12	19	0.40	0.05, 0.75
*13*	*31*	*0.52*	*0.27, 0.78*
14	31	0.19	-0.08, 0.47
*15*	*19*	*0.61*	*0.28, 0.94*
16	31	0.34	0.06, 0.63

Italicised questions are considered critical by the tool authors

##### Summary of the findings on Inter-rater reliability

**Fig. 1:**
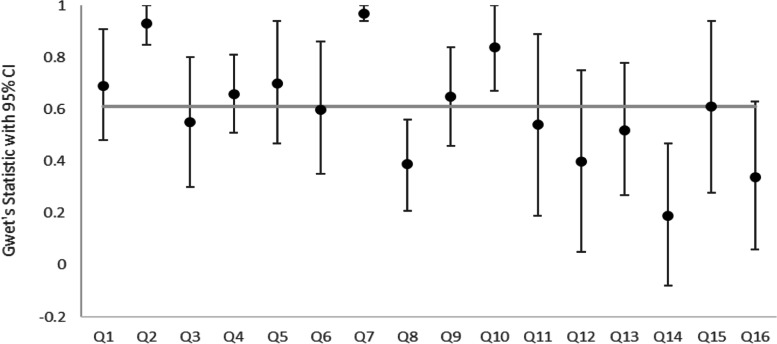
Gwet’s statistic for the inter-rater agreement for AMSTAR-2 questions

In total, 460 comparisons were included in the analysis for AMSTAR-2. The median agreement for all questions was 0.61. Eight of the 16 AMSTAR-2 questions had substantial agreement or higher. There was almost perfect agreement for questions 2 (did the report of the review contain an explicit statement that the review methods were established prior to the conduct of the review and did the report justify any significant deviations from the protocol?), 7 (did the review authors provide a list of excluded studies and justify the exclusions?) and 10 (did the review authors report on the sources of funding for the studies included in the review?). The lowest agreement was for question 14 (did the review authors provide a satisfactory explanation for, and discussion of, any heterogeneity observed in the results of the review?). Ratings were missing in 35 cases. The results are displayed in Fig. 1.

The AMSTAR-2 critical questions, in particular, seemed to have good agreement compared to the other questions. There was at least substantial agreement for all critical questions except question 13 which had moderate agreement. Questions 2 and 7 both had almost perfect agreement and had the highest agreement of all AMSTAR-2 questions.

Gwet’s AC2 statistic was used for questions 2, 4, 7, 8 and 9. Gwet’s AC1 statistic was used for all other questions. The markers represent the Gwet’s statistic and the error bars represent the 95% confidence intervals. The italicised data represent the median value for all questions.

Further information on the separate reviews can be found in the Appendix (Tables 7 and 11). The overall median IRR agreement for AMSTAR-2 questions for fibromyalgia is 0.65 and for colic is 0.60.

#### ROBIS

##### Summary of the ROBIS results

The consensus results for ROBIS for both fibromyalgia and colic overviews can be found in Table 4. With regard to the ROBIS results, domain 1 (which assessed any concerns regarding specification of study eligibility criteria), 9 fibromyalgia reviews achieved a low risk of bias rating overall and 6 colic reviews achieved a low risk of bias rating overall. In domain 2 (which assessed concerns regarding methods used to identify and/or select studies), 7 fibromyalgia reviews achieved a low risk of bias rating overall and 6 colic reviews achieved a low risk of bias rating overall.

**Table 4:** Tabular presentation for agreement of ROBIS results

**Fibromyalgia review**	**Phase 2**				**Phase 3**
	**1. Study eligibility criteria**	**2. Identification and selection of studies**	**3. Data collection and study appraisal**	**4. Synthesis and findings**	**5. Risk of bias in the review**
**Homoeopathy**					
1. Perry	Low	Low	Low	Unclear	Low
2. Boehm	High	Low	Low	High	High
**Acupuncture**					
3. Mayhew	Low	High	High	Low	Low
4. Daya	Low	High	High	Low	Low
5. Langhorst	Low	High	High	Low	Low
6. Martin-Sanchez	Low	High	High	High	High
7. Cao	Low	High	Low	Low	Low
8. Deare	Low	Low	Low	Low	Low
9. Yang	Low	Low	High	High	High
**Chiropractic**					
10. Ernst	High	Unclear	High	Unclear	Unclear
**Herbal Medicine**					
11. Nascimento	Low	Low	Low	High	Low
**Multiple CAM reviews**					
12. Holdcraft	Low	Low	Low	High	Low
13. Baronowsky	Low	Low	Unclear	High	Low
14. Terhorst	Low	High	Low	High	High
15. De Silva	High	High	High	Unclear	Low
**Colic review**	**Phase 2**				**Phase 3**
	**1. Study eligibility criteria**	**2. Identification and selection of studies**	**3. Data collection and study appraisal**	**4. Synthesis and findings**	**5. Risk of bias in the review**
**Multiple CAM therapies**					
1. Perry	Low	Unclear	Low	Low	Low
2. Bruyas-Bertholon	High	High	Unclear	High	High
3. Harb	High	High	Low	High	High
4. Gutierrez-Castrellon	Unclear	High	High	High	High
**Manipulation therapies**					
5. Dobson	Low	Low	Low	Low	Low
6. Gleberzon	High	High	Unclear	Unclear	High
7. Carne	Low	Low	Low	High	Unclear
**Acupuncture**					
8. Skejeie	Low	Low	Low	Low	Unclear
**Herbal medicine**					
9. Anheyer	Unclear	High	Low	High	High
**Probiotics**					
10. Sung 2013	Unclear	Low	Low	High	Unclear
11. Anabrees	Low	Low	Low	High	Low
12. Urbansk	Low	High	High	High	High
13. Xu	Unclear	Low	Low	Unclear	Low
14. Shreck Bird	High	High	Low	High	High
15. Dryl	High	High	Unclear	High	High
16. Sung 2018	High	Unclear	Unclear	Unclear	Unclear

Domain 3 assessed concerns regarding methods used to collect data and appraise studies; 7 fibromyalgia studies and 10 colic reviews achieved a low risk of bias rating overall.

With regard to domain 4 (which assessed concerns regarding the synthesis and findings), more variation in the fibromyalgia scores was found, whereas most colic reviews were rated as high risk of bias in this domain. The reviews that did not conduct a meta-analysis were hard to assess using ROBIS.

The final section provides a rating for the overall risk of bias of the reviews; 7 fibromyalgia reviews achieved a low rating; 6, a high rating; and 2, were rated as unclear. Four colic reviews achieved a low rating; 4, an unclear rating; and 8, a high rating.

##### Results of inter-rater reliability analysis for ROBIS

A summary of the inter-rater reliability for ROBIS can be found in Table 5.

**Table 5:** Inter-rater agreement

ROBIS question	No. of studies	Gwet’s AC1/Gwet’s AC2	95% CI
**Domain 1: study eligibility criteria**			
1.1	30	0.62	0.38, 0.85
1.2	31	0.70	0.56, 0.84
1.3	31	0.69	0.56, 0.82
1.4	31	0.61	0.48, 0.74
1.5	31	0.56	0.37, 0.74
**Domain 1** Concerns regarding specification of study eligibility criteria	31	0.45	0.22, 0.67
**Domain 2: identification and selection of studies**			
2.1	31	0.53	0.41, 0.65
2.2	30	0.53	0.35, 0.71
2.3	31	0.62	0.47, 0.77
2.4	31	0.41	0.20, 0.62
2.5	29	0.59	0.30, 0.88
**Domain 2** Concerns regarding methods used to identify and/or select studies	31	0.36	0.17, 0.55
**Domain 3: data collection and study appraisal**			
3.1	29	0.88	0.68, 1.00
3.2	31	0.66	0.51, 0.82
3.3	31	0.65	0.51, 0.78
3.4	31	0.77	0.61, 0.93
3.5	30	0.73	0.48, 0.98
**Domain 3** Concerns regarding methods used to collect data and appraise studies	31	0.55	0.35, 0.76
**Domain 4: synthesis and findings**			
4.1	31	0.60	0.46, 0.74
4.2	29	0.48	0.28, 0.68
4.3	31	0.77	0.66, 0.88
4.4	31	0.18	− 0.02, 0.37
4.5	30	0.22	0.02, 0.43
4.6	31	0.39	0.17, 0.62
**Domain 4** Concerns regarding the synthesis and findings	31	0.17	− 0.03, 0.37
**Risk of bias in the review**			
A	31	0.28	0.09, 0.47
B	31	0.64	0.54, 0.75
C	31	0.45	0.31, 0.60
**ROB**	31	0.45	0.24, 0.66

##### Summary of the findings on Inter-rater reliability

**Fig. 2:**
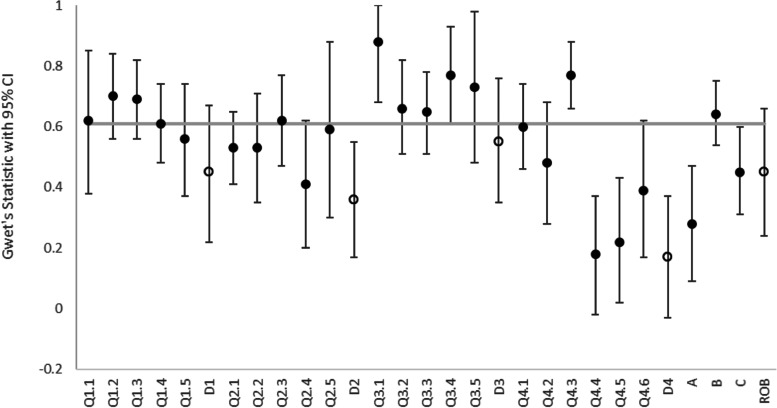
Gwet’s statistic for the inter-rater agreement for ROBIS questions and domains

For ROBIS, there were 734 comparisons considered for the 24 questions. The median agreement for all questions was 0.61. Eleven of the 24 ROBIS questions had substantial agreement or higher. Ratings were missing in 9 cases. At least one rater said “no information” in 159 comparisons. Rater 1 used “no information” 73 times; rater 2, 50 times; and rater 3, 93 times. In 107 comparisons only one rater said “no information” and the raters all agreed only in 10 comparisons. “No information” was used most frequently for question 1.1 (did the review adhere to pre-defined objectives and eligibility criteria? 23 studies), question 4.2 (were all pre-defined analyses reported or departures explained? 22 studies) and question 4.5 (were the findings robust, e.g., as demonstrated through funnel plot or sensitivity analyses? 16 studies). The agreement was “moderate” for domains 1 (0.45) and 3 (0.36) and for the overall risk of bias (0.45). The agreement for domains 2 and 4 were “fair” (0.36) and “slight” (0.17), respectively. The results are summarised in Fig. 2.

Gwet’s AC2 statistic was used for the ROBIS questions (filled markers) and Gwet’s AC1 statistic was used for the ROBIS domains (hollow markers). The error bars represent the 95% confidence intervals. The italicised data represent the median value for all ROBIS questions.

Further information on the separate reviews can be found in the appendix (Tables 8 and 12). The median IRR agreement for all ROBIS questions for FM is 0.55 and for colic is 0.63.

### Section 3: Usability of the tools

All three raters felt AMSTAR-2 was more straightforward and user-friendly than ROBIS. This might be because it does not require expertise in systematic reviewing to complete this tool, just knowledge of trial design.

Several issues arose from using the ROBIS tool as it required more consideration to complete. Within each domain, each question had five possible responses (yes, probably yes, probably no, no, no information), although at times it was difficult to distinguish between yes/probably yes and no/probably no. It also might be more helpful to have a choice of “no concerns/minor concerns/ major concerns/considerable concerns”, instead of “low/high/unclear” judgements that are currently at the end of each domain when assessing the overall judgement of concerns. Although there were perceived differences in the individual answers to each signalling question between reviewers, the overall rating of the domains was more consistent. Overall, domains 1–3 were easier to follow and score.

The most difficult domain to score was domain 4 which covers “synthesis of evidence”. This was reflected in the lowest agreement between raters (0.17). We found that this domain is currently better designed for a review with a meta-analysis, rather than a narrative synthesis. The guidance document that accompanies the tool is long and difficult to navigate. On the plus side, despite having subjective opinions (within each domain there was variation between the reviewers’ responses to the signalling questions), you can still end with a moderately consistent overall result (0.45).

The ROBIS tool provides an overall sense of risk of bias of the review. There is better coverage overall than AMSTAR-2 and more precision with the use of a final rating. From our observations only, higher quality reviews were quicker to appraise. In our analysis, the “no information” rating for ROBIS questions was treated as missing. The raters rarely agreed on when to use this rating. In most cases, when one rater reported “no information” for a ROBIS question, the other two raters gave a different rating.

Several issues arose from using AMSTAR-2. Sometimes, the raters would have opted for a “partially yes” option when only a binary option (yes/no) was available (Q13, Q14, Q16). Also, some questions were ambiguous; in particular, Q3 asks if authors explain their selection of study design (e.g., use of RCTs/non RCTs); some reviews merely report they included RCTs rather than justifying their selection, which caused discrepancies between raters.

Also, some questions might elicit a different response depending on the outcome, e.g., Q13 (whether risk of bias was discussed/interpreted within the results), which may vary depending on whether there were multiple outcomes, and thus, which outcome is being referred to.

The raters also felt it would be helpful to have a formal space to add comments to justify their decision to help with discussions, as in the more ambiguous reviews; decisions were more open to interpretation. ROBIS, on the other hand, has a large section where the reviewer is expected to add selected text to support their decision.

Regarding completion timings, we were able to establish how long it took to complete both tools for one of the overviews (colic). There was little difference in timings between rater 1 and 2 to complete both tools; in fact, it took rater 2 slightly longer to complete AMSTAR-2 than ROBIS which is surprising, considering the issues reported above. However, rater 3 took considerably longer to complete ROBIS than AMSTAR-2 (see Table 6).

**Table 6:** Mean (SD) completion time (in minutes) for colic paper

	Rater 1		Rater 2		Rater 3	
	*n*	Mean (SD)	*n*	Mean (SD)	*n*	Mean (SD)
**AMSTAR-2**	14	13.0 (5.2)	15	18.7 (6.6)	16	11.1 (4.2)
**ROBIS**	9	14.1 (6.5)	10	15.7 (5.3)	15	43.3 (23.3)

Rater 3 was the most experienced reviewer and helped develop the ROBIS tool. They spent longer on bringing the evidence forward from the individual reviews into the ROBIS extraction form as recommended by the guidance document, whereas the other two raters only wrote cursory notes.

It is important to highlight that it is advised in the ROBIS guidance document that it is a tool aimed at experienced systematic reviewers and methodologists. We would agree with this recommendation but recognise that this is not often the case in many groups undertaking reviews.

## Discussion

### Summary of findings

The median inter-rater reliability (IRR) agreement for both AMSTAR-2 and ROBIS questions was substantial: 50% of AMSTAR-2 questions and 46% of ROBIS questions had substantial agreement or higher. For AMSTAR-2, 460 comparisons were included in the analysis. The median agreement for all questions was 0.61. For ROBIS, there were 734 comparisons considered for the 24 questions. The median agreement for all questions was also 0.61. It is interesting that the median IRR agreement for both tools was 0.61, demonstrating a similar level of rating between the two scales.

Results were similar when conducting the analysis for fibromyalgia and colic reviews separately (see appendix for independent overview results). For fibromyalgia, the median IRR value was 0.66 for the AMSTAR-2 questions compared to 0.56 for the ROBIS questions. For the colic studies both AMSTAR-2 and ROBIS had a similar median (0.60 for AMSTAR-2 and 0.63 for ROBIS).

It must also be considered that the ROBIS questions include more categories than most of the AMSTAR-2 questions. Most AMSTAR-2 questions are binary. Inter-rater agreement tends to be lower when there are more categories, as there are more possibilities for disagreement. Similarly, ROBIS includes more questions than AMSTAR-2 which can also result in more disagreement. However, despite these differences, the median agreement was the same for the AMSTAR-2 and ROBIS questions.

### Usability of the tools

Several issues arose when using the ROBIS tool as it required more consideration to complete, which could become problematic in a large review. All three raters felt AMSTAR-2 was more straightforward and user-friendly than ROBIS. This might be because it does not require expertise in systematic reviewing to complete this tool, just knowledge of trial design.

AMSTAR-2 was considered quicker to work through than ROBIS, yet the median timings demonstrated only a slight increase in timing on AMSTAR-2 than ROBIS in two raters, although one rater did take considerably longer on ROBIS than AMSTAR-2. All raters felt domain 4 of ROBIS was particularly difficult to complete if there was no meta-analysis. Domain 4 would benefit from further development in order to assess reviews without a meta-analysis, as in some ways it is biassed against these types of reviews.

### Relationship to background research

Previous research [7, 8] compared four raters’ assessments across 30 systematic reviews. They calculated the IRR using the Fleiss’ *k* [45]. The IRR for scoring the overall confidence in the SRs with AMSTAR-2 was fair (AMSTAR-2: *κ* = 0.30; 95% [confidence interval] CI, 0.17 to 0.43). The overall domain in ROBIS was fair (ROBIS: κ = 0.28; 95% CI, 0.13 to 0.42). Interestingly, for the overall rating, AMSTAR-2 showed a high concordance with ROBIS and a lower concordance with AMSTAR.

We were unable to directly compare our results against Pieper’s work, as the Fleiss’ kappa ignores the order of the categories (when there are more than two categories), which is why we used Gwet’s as it takes the order into account and allows for “partial agreement”. Also, Gwet scores tend to be higher than Fleiss scores in general, which makes comparisons difficult to conduct.

In Pieper et al.’s [7] study, ROBIS was always applied after AMSTAR-2, and the mean time for scoring AMSTAR-2 was slightly higher than for ROBIS (18 vs. 16 min), with huge variation between the reviewers, whereas in our study, the overall mean time (calculated for colic reviews only) was slightly higher for ROBIS than for AMSTAR-2 (24.4 min compared to 14.3 min), although the mean ROBIS result was largely influenced by one rater.

### Potential bias in the overview process

One author evaluated their own work using AMSTAR-2 and ROBIS (RP: [19, 29]), although this work was also independently assessed by two other reviewers (VL, PD). In addition, one of the developers of ROBIS (PD) applied the ROBIS tool to assess the included reviews.

We had not planned to complete an IRR assessment of the two scales whilst completing these two overviews of reviews; therefore, we did not apply strict criteria to our assessment schedule, i.e., we did not apply the tools in any particular order. We also did not complete timings for some of our assessments in a systematic way.

Another issue is we compared our ratings over time, i.e., a batch of five papers were discussed before the next batch was assessed; this is likely to have led to greater consistency between the raters over time, but our numbers were too small to check this.

## Conclusion

In terms of quality assessment, ROBIS is an effective tool for assessing risk of bias in a systematic review but is more difficult to use compared to AMSTAR-2. It is more complex to work through, which might be problematic in a large review. As suggested by the developers of ROBIS; it is best used by experienced systematic reviewers/methodologists. Reviews that included a meta-analysis were easier to rate, however, further developmental work could improve its use in systematic reviews without a meta-analysis. AMSTAR-2 was more user-friendly and was effective at measuring quality of a review but was a less sophisticated tool. Both tools could do with minor changes to help improve their useability for people conducting systematic reviews.

## Data Availability

Not relevant.

## Appendix

### Results of AMSTAR-2 for CAM for fibromyalgia reviews

The inter-rater agreement between the three raters is shown in Table 7.

**Table 7:** Results of AMSTAR-2 for CAM for fibromyalgia reviews

Question	No. of studies	Gwet’s AC1/Gwet’s AC2	95% CI	***p***-value
1	15	0.66	0.32, 1.00	0.001
*2*	*15*	*1.00*		
3	15	0.39	− 0.08, 0.86	0.096
*4*	*15*	*0.74*	*0.55, 0.93*	*< 0.001*
5	15	0.69	0.33, 1.00	0.001
6	15	0.65	0.26, 1.00	0.003
*7*	*15*	*1.00*		
8	15	0.20	0.02, 0.38	0.031
*9*	*15*	*0.37*	*0.16, 0.59*	*0.002*
10	15	1.00	0.85, 1.00	< 0.001
*11*	*7*	*0.66*	*0.01, 1.00*	*0.047*
12	7	0.52	− 0.11, 1.00	0.091
*13*	*15*	*0.62*	*0.26, 0.98*	*0.002*
14	15	0.20	− 0.17, 0.57	0.270
*15*	*7*	*0.70*	*0.10, 1.00*	*0.029*
16	15	0.55	0.14, 0.96	0.013

Twenty missing ratings. Italicised areas are considered the critical questions

### Results of ROBIS: CAM for fibromyalgia

The summary of results of ROBIS for fibromyalgia can be seen in Table 8.

**Table 8:** Inter-rater agreement

ROBIS question	No. of studies	Gwet’s AC1/Gwet’s AC2	95% CI	***p***-value
**Domain 1: study eligibility criteria**				
1.1	14	0.73	0.46, 1.00	< 0.001
1.2	15	0.70	0.45, 0.95	< 0.001
1.3	15	0.62	0.39, 0.84	< 0.001
1.4	15	0.54	0.32, 0.76	< 0.001
1.5	15	0.64	0.40, 0.88	< 0.001
***Domain 1****Concerns regarding specification of study eligibility criteria*	*15*	*0.61*	*0.29, 0.92*	*0.001*
**Domain 2: identification and selection of studies**				
2.1	15	0.53	0.36, 0.69	< 0.001
2.2	14	0.42	0.16, 0.69	0.005
2.3	15	0.72	0.53, 0.92	< 0.001
2.4	15	0.31	− 0.08, 0.70	0.110
2.5	15	0.56	0.14, 0.99	0.013
***Domain 2****Concerns regarding methods used to identify and/or select studies*	*15*	*0.29*	*0.03, 0.55*	*0.031*
**Domain 3: data collection and study appraisal**				
3.1	15	0.95	0.66, 1.00	< 0.001
3.2	15	0.65	0.47, 0.84	< 0.001
3.3	15	0.57	0.40, 0.74	< 0.001
3.4	15	0.55	0.23, 0.88	0.003
3.5	15	0.81	0.51, 1.00	< 0.001
***Domain 3****Concerns regarding methods used to collect data and appraise studies*	*15*	*0.52*	*0.19, 0.83*	*0.004*
**Domain 4: synthesis and findings**				
4.1	15	0.55	0.33, 0.77	< 0.001
4.2	13	0.55	0.29, 0.81	0.001
4.3	15	0.80	0.62, 0.98	< 0.001
4.4	15	0.13	− 0.19, 0.45	0.405
4.5	14	− 0.10	− 0.52, 0.33	0.633
4.6	15	0.23	− 0.17, 0.64	0.235
***Domain 4****Concerns regarding the synthesis and findings*	*15*	*0.18*	*− 0.08, 0.44*	*0.154*
**Risk of bias in the review**				
A	15	0.10	− 0.25, 0.44	0.552
B	15	0.61	0.40, 0.83	< 0.001
C	15	0.39	0.01, 0.76	0.009
***ROB***	*15*	*0.43*	*0.10, 0.77*	*0.015*

Six ratings missing

### Inter-rater agreement for fibromyalgia

For AMSTAR-2, 10 out of 16 (62.5%) questions had substantial agreement or higher between reviewers. There was perfect agreement for questions 2 (did the report of the review contain an explicit statement that the review methods were established prior to the conduct of the review and did the report justify any significant deviations from the protocol?), 7 (did the review authors provide a list of excluded studies and justify the exclusions?) and 10 (did the review authors report on the sources of funding for the studies included in the review?). The median agreement for all questions was 0.65. Ratings from a reviewer were missing in 20 instances overall.

Ten out of 24 (41.7%) questions for ROBIS had at least substantial agreement. Questions 3.1 (were efforts made to minimise error in data collection?) and 3.5 (were efforts made to minimise error in risk of bias assessment?) had almost perfect agreement. The median agreement for all questions was 0.55. The agreement was different for each ROBIS domain with substantial being the highest agreement (for missing in 6 instances). The raters gave a rating of “no information” in 93 cases. In most of these cases (65), the other two raters gave a different rating. There were 5 instances where all reviewers reported “no information”. The most common questions for “no information” were questions 1.1 (did the review adhere to pre-defined objectives and eligibility criteria? 13 times), 4.2 (were all pre-defined analyses reported or departures explained? 13 times) and 4.5 (were the findings robust, e.g. as demonstrated through funnel plot or sensitivity analyses? 11 times) .

Tables 9 and 10

**Table 9:** The risk of bias and study quality for each fibromyalgia review

Fibromyalgia	AMSTAR-2	ROBIS
**Multiple CAM therapies**		
*Holdcraft 2003* [14]	***CL***	***Low***
Baronowsky 2009 [15]	**CL**	**High**
Terhorst 2011, 2012 [16, 17]	**CL**	**High**
De Silva 2010 [18]	**CL**	**High**
**Homoeopathy**		
*Perry 2010* [19]	***CL***	***Low***
Boehm 2014 [20]	**CL**	**High**
**Chiropractic treatment**		
Ernst 2009 [21]	**CL**	**Unclear**
**Acupuncture**		
*Mayhew and Ernst 2007* [22]	***CL***	***Low***
*Daya 2007* [23]	***CL***	***Low***
*Langhorst 2010* [24]	***CL***	***Low***
Martin-Sanchez 2009 [25]	**CL**	**High**
*Cao 2013* [26]	***CL***	***Low***
Deare 2013 [13]	**LOW**	**Low**
Yang 2014 [27]	**CL**	**High**
**Herbal medicines**		
*de Souza Nascimento 2013* [28]	***CL***	***Low***

When AMSTAR-2 is low, this should correspond to ROBIS being of high risk of bias. The italicised reviews show discrepancies between the overall rating of quality/bias

**Table 10:** To compare the distribution of risk of bias and study quality for the fibromyalgia reviews

ROBIS				
**AMSTAR-2**		**High**	**Low**	**Unclear**
	**High**	**0**	**0**	**0**
	**Moderate**	**0**	**0**	**0**
	**Low**	**0**	**1**	**0**
	**Critical**	**6**	**7**	**1**

### Results of AMSTAR-2: CAM for colic

The inter-rater agreement between the three raters is shown in Table 11.

**Table 11:** Inter-rater agreement

Question	No. of studies	Gwet’s AC1/Gwet’s AC2	95% CI	***p***-value
1	16	0.73	0.43, 1.00	< 0.001
*2*	*16*	*0.83*	*0.64, 1.00*	*< 0.001*
3	16	0.68	0.40, 0.96	< 0.001
*4*	*16*	*0.58*	*0.34, 0.83*	*< 0.001*
5	16	0.72	0.38, 1.00	< 0.001
6	16	0.56	0.18, 0.95	0.006
*7*	*16*	*0.91*	*0.81, 1.00*	*< 0.001*
8	16	0.61	0.35, 0.87	< 0.001
*9*	*16*	*0.87*	*0.69, 1.00*	*< 0.001*
10	16	0.67	0.36, 0.97	< 0.001
*11*	*12*	*0.49*	*0.02, 0.96*	*0.042*
12	12	0.34	− 0.12, 0.80	0.133
*13*	*16*	*0.43*	*0.03, 0.84*	*0.038*
14	16	0.22	− 0.23, 0.66	0.321
*15*	*12*	*0.58*	*0.16, 0.99*	*0.011*
16	16	0.15	− 0.25, 0.55	0.444

Fifteen missing ratings. Italicised areas are considered the critical questions

### Results of ROBIS: CAM for colic

The inter-rater agreement between the three raters is shown in Table 12

**Table 12:** Inter-rater agreement

ROBIS question	No. of studies	Gwet’s AC1/Gwet’s AC2	95% CI	***p***-value
**Domain 1: study eligibility criteria**				
1.1	16	0.57	0.17, 0.96	0.008
1.2	16	0.71	0.55, 0.87	< 0.001
1.3	16	0.76	0.61, 0.91	< 0.001
1.4	16	0.71	0.54, 0.87	< 0.001
1.5	16	0.49	0.20, 0.77	0.002
***Domain 1****Concerns regarding specification of study eligibility criteria*	*16*	*0.30*	*− 0.03, 0.63*	*0.072*
**Domain 2: identification and selection of studies**				
2.1	16	0.54	0.34, 0.73	< 0.001
2.2	16	0.64	0.37, 0.92	< 0.001
2.3	16	0.57	0.34, 0.81	< 0.001
2.4	16	0.50	0.27, 0.73	< 0.001
2.5	14	0.61	0.18, 1.00	< 0.001
***Domain 2****Concerns regarding methods used to identify and/or select studies*	*16*	*0.43*	*0.13, 0.73*	*0.008*
**Domain 3: data collection and study appraisal**				
3.1	14	0.82	0.51, 1.00	< 0.001
3.2	16	0.70	0.44, 0.96	< 0.001
3.3	16	0.72	0.52, 0.92	< 0.001
3.4	16	0.92	0.83, 1.00	< 0.001
3.5	15	0.66	0.21, 1.00	0.007
***Domain 3****Concerns regarding methods used to collect data and appraise studies*	*16*	*0.61*	*0.32, 0.89*	*< 0.001*
**Domain 4: synthesis and findings**				
4.1	16	0.65	0.45, 0.86	< 0.001
4.2	16	0.42	0.11, 0.73	0.011
4.3	16	0.73	0.58, 0.88	< 0.001
4.4	16	0.23	− 0.02, 0.48	0.072
4.5	16	0.40	0.22, 0.57	< 0.001
4.6	16	0.55	0.32, 0.77	< 0.001
***Domain 4****Concerns regarding the synthesis and findings*	*16*	*0.17*	*− 0.17, 0.50*	*0.305*
**Risk of bias in the review**				
A	16	0.47	0.28, 0.65	0.015
B	16	0.69	0.55, 0.82	< 0.001
C	16	0.54	0.37, 0.72	< 0.001
***ROB***	*16*	*0.47*	*0.17, 0.77*	*0.004*

Three ratings missing

### Inter-rater agreement for colic

Eight of 16 (50%) AMSTAR-2 questions had substantial agreement or higher. There was almost perfect agreement for questions 2 (did the report of the review contain an explicit statement that the review methods were established prior to the conduct of the review and did the report justify any significant deviations from the protocol?), 7 (did the review authors provide a list of excluded studies and justify the exclusions?) and 9 (did the review authors use a satisfactory technique for assessing the risk of bias (RoB) in individual studies that were included in the review?). The median score for all questions was 0.60. Ratings from a reviewer were missing in 15 instances overall.

Thirteen of 24 (54.2%) ROBIS questions had substantial agreement or higher. There was almost perfect agreement for questions 3.1 (were efforts made to minimise error in data collection?) and 3.4 (was risk of bias (or methodological quality) formally assessed using appropriate criteria?). The median score for all questions was 0.63. The agreement was different for each ROBIS domain with substantial being the highest agreement (for domain 3). The agreement for the risk of bias was moderate. Ratings from a reviewer were missing in 3 instances. There were 66 ratings of “no information”. There were 3 instances where the reviewers were in agreement. In 42 cases, only one reviewer said “no information”. The most common questions were questions 1.1 (did the review adhere to pre-defined objectives and eligibility criteria? 10 times), 3.5 (were efforts made to minimise error in risk of bias assessment? 8 times) and 4.2 (were all pre-defined analyses reported or departures explained? 9 times).

Tables 13, 14 and 15

**Table 13:** The risk of bias and study quality for each colic review

Colic	AMSTAR-2	ROBIS
**Multiple CAM therapies**		
*Perry 2011* [29]	***Low***	***Low***
Bruyas-Bertholon 2012 [30]	**CL**	**High**
Harb 2016 [31]	**CL**	**High**
Gutierrez-Castrellon 2017 [32]	**CL**	**High**
**Manipulation therapies**		
Dobson 2012 [33]	**High**	**Low**
Gleberzon 2012 [34]	**CL**	**High**
Carnes 2017 [35]	**CL**	**Unclear**
**Acupuncture**		
Skejeie 2018 [36]	**Low**	**Unclear**
**Herbal medicine**		
Anheyer 2017 [37]	**CL**	**High**
**Probiotics**		
Sung 2013 [38]	**CL**	**Unclear**
*Anabrees 2013* [39]	***Low***	***Low***
Urbanska 2014 [40]	**CL**	**High**
*Xu 2015* [41]	***CL***	***Low***
Schreck Bird 2017 [42]	**CL**	**High**
Dryl 2018 [43]	**CL**	**High**
Sung 2018 [44]	**LOW**	**Unclear**

When AMSTAR-2 is low, this should correspond to ROBIS being of high risk of bias. The italicised reviews show discrepancies between the overall rating of quality/bias

**Table 14:** To compare the distribution of risk of bias and study quality for the fibromyalgia reviews

ROBIS				
**AMSTAR-2**		**High**	**Low**	**Unclear**
	**High**	**0**	**1**	**0**
	**Moderate**	**0**	**0**	**0**
	**Low**	**0**	**2**	**1**
	**Critical**	**8**	**1**	**3**

**Table 15:** Criteria for assessing confidence in AMSTAR-2 (Shea et al. [20])

Rating overall confidence in the results of the review	
1. **High** (a) *No or one non-critical weakness*. The systematic review provides an accurate and comprehensive summary of the results of the available studies that address the question of interest 2. **Moderate** (a) *More than one non-critical weakness**. The systematic review has more than one weakness but no critical flaws. It may provide an accurate summary of the results of the available studies that were included in the review 3. **Low** (a) *One critical flaw with or without non-critical weaknesses*. The review has a critical flaw and may not provide an accurate and comprehensive summary of the available studies that address the question of interest 4. **Critically low** (a) *More than one critical flaw with or without non-critical weaknesses*. The review has more than one critical flaw and should not be relied on to provide an accurate and comprehensive summary of the available studies	

*Multiple non-critical weaknesses may diminish confidence in the review and it may be appropriate to move the overall appraisal down from moderate to low confidence

## Abbreviations

CAM: Complementary and alternative medicine
AMSTAR: Assessment of Multiple Systematic Reviews
PICO: Population, intervention, control group, outcome
ROBIS: Risk of Bias in Systematic reviews
IRR: Inter-rater reliability
